# Analysis of urinary volatile organic compounds for prostate cancer diagnosis: A systematic review

**DOI:** 10.1002/bco2.423

**Published:** 2024-08-06

**Authors:** Jonathon Dawson, Kraig Green, Henry Lazarowicz, Phil Cornford, Chris Probert

**Affiliations:** ^1^ University of Liverpool UK; ^2^ Liverpool University Hospitals NHS Foundation Trust UK

**Keywords:** diagnostics, eNose, gas chromatography mass spectrometry, metabolomics, prostate cancer, volatile organic compounds

## Abstract

**Context:**

Prostate‐specific antigen is non‐specific for prostate cancer. This is improved by multiparametric MRI but a significant amount of indolent prostate cancer is detected by the current MRI pathway and data is emerging that clinically significant cancers maybe missed using a standard PSA threshold. Volatile organic compound (VOC) analysis may offer novel biomarkers for prostate cancer and clinically significant disease.

**Objective:**

To perform a systematic review of the literature to evaluate the current evidence for the use of VOCs as novel biomarkers for prostate cancer and clinically significant prostate cancer.

**Evidence Acquisition:**

A systematic search of MEDLINE, Scopus, Web of Science and the Cochrane Library was undertaken by two independent reviewers and papers were assessed for inclusion in the review. Study characteristics, sensitivity and specificity of GC–MS or eNose were extracted. Risk of bias and applicability issues were determined using QUADAS 2 and the quality of reporting using the STARD checklist.

**Evidence Synthesis:**

Nineteen studies were included, of which 6 utilised eNose and 13 GC–MS. eNose sensitivity and specificity were 0.71–0.95 and 0.79–0.96, respectively, and GC–MS found a sensitivity and specificity of 0.66–1.00 and 0.53–0.97, respectively. There were concerns about bias in patient recruitment due to differences in the timing of the index test relative to the reference standard.

**Conclusion:**

This review has found promising early results for urinary metabolomics in the detection of prostate cancer. However, there is a need for larger, high‐quality studies to validate this. Future work should focus on the detection of clinically significant prostate cancer.

## 1 INTRODUCTION

Prostate cancer (PCa) is the most common cancer in males in the United Kingdom, with an estimated annual incidence of 52 200 cases.^1^ Incidence has increased steadily since the discovery of prostate‐specific antigen (PSA) in 1978.^1^, ^2^ The current diagnostic pathway for patients with suspected PCa includes a clinical assessment with digital rectal examination and a PSA test. Patients with abnormal results are risk stratified with multiparametric MRI (mpMRI) and if there is a subsequent suspicion of PCa an ultrasound‐guided biopsy is performed.^3^ These investigations are expensive, potentially morbid and require highly‐trained clinicians to perform and interpret the results.

PSA can be elevated in various prostate pathologies including benign prostatic hyperplasia (BPH), infection or following trauma to the prostate, for example from catheterisation or digital rectal examination, and is therefore non‐specific for prostate cancer. A PSA > 4 ng/ml has a positive predictive value of just 25%.^4^ Its widespread use, therefore, contributes to the over‐investigation of men without PCa, resulting in psychological distress and a burden on healthcare systems.

When used in screening studies, PSA was found to increase prostate cancer detection but did not improve overall survival or cancer‐specific survival compared to unscreened controls.^5^ This illustrates the inability of PSA to discriminate between patients with indolent diseases compared to those with clinically significant PCa (csPCa) who require radical treatment to prevent disease progression and its associated morbidity and mortality.

The main challenges in PCa diagnostics are therefore two‐fold: firstly, to improve the specificity of investigations at initial workup, to reduce the burden of unnecessary investigation on patients and health systems; and secondly, to select out those who are unlikely to require treatment due to the presence of indolent disease.

Multiple urine‐based biomarkers have been trialled, largely based on urinary nucleosome genomic analysis, but so far none have been validated in large‐scale studies.^6^ Another emerging area of study for biomarker identification is urinary metabolomics. Volatile organic compounds (VOCs) are a heterogeneous group of short carbon chain metabolites, of which 444 have been identified in human urine.^7^ Urine represents an ideal matrix for the study of VOC profiles in PCa as it is easy to collect and is acceptable to patients, contains many potential biomarkers and the anatomical location of the prostate in the urinary tract increases the likelihood of VOCs being excreted directly into the urine.

Early studies utilising canine olfaction for the discrimination of urine from patients with PCa from benign control groups showed promising results, with sensitivities and specificities of 95–100%.^8^ However, these studies were limited by several factors. They were relatively small and unblinded, so at risk of recruitment bias. Additionally, only a small number of dogs were tested, so it is possible there may not have been a high degree of agreement between dogs. This was demonstrated in a study of four dogs, with the best‐performing dog giving a specificity of 92%, and the worst 56%.^9^ Dogs are not classified as medical devices and so there may be concerns regarding regulatory processes. However, these canine studies raised interest in the possibilities.

Advances in metabolomic science have enabled different analytical modalities including gas chromatography–mass spectroscopy (GC–MS) and semiconductor sensor‐based devices (also known as eNoses) to search for novel biomarkers of PCa. To increase the number of compounds released into the headspace for detection, the ionic strength or pH of the sample can be adjusted with salt, acid or alkali. The compounds detected vary depending on the type and concentration of chemical pre‐treatment.^10^ GC–MS is considered the gold standard because it allows the identification of individual metabolites and therefore offers the potential for a deeper understanding of the metabolic differences between disease and health. eNoses, however, are technically easier to use and may be more practical for untrained users, so may offer more scope for scalability in day‐to‐day clinical use. Nuclear magnetic resonance (NMR) spectroscopy is unlikely to be applicable to most clinical laboratory settings due to cost and logistical implications and will therefore not be included in the literature search.

This review assesses the current state of metabolomic‐based PCa diagnostics. Firstly, with a systematic search of the available literature assessing the accuracy of GC–MS and eNose in the diagnosis of PCa, critically appraising the methodology, quality of reporting and risk of bias in each paper and finally presenting recommendations for future directions of research.

## 2 EVIDENCE ACQUISITION

The protocol for this review was registered with PROSPERO (ID CRD42023405598). A structured search was undertaken using MEDLINE, Scopus, Web of Science and the Cochrane Library for English language peer‐reviewed journal articles from 01/01/2010 to 01/03/2023 inclusive. Search terms included truncated terms and synonyms for words related to PCa, diagnostic accuracy, GC–MS and eNose (See Figure S1). Databases were searched on 07/03/2023. Two independent reviewers conducted searches and eligibility screening separately.

Following deduplication, titles and abstracts were screened for potentially relevant content. Once screened, the remaining manuscripts were obtained, and each author separately composed a preliminary list of studies meeting the inclusion criteria. Authors were unblinded to each other's selections, lists were compared, and any discrepancies were resolved by consensus. The outcome of the screening process was recorded and displayed according to the Preferred Reporting Items for Systematic Reviews and Meta‐Analyses (PRISMA) guidelines.

Eligibility criteria for studies to be included in the final analysis included any study reporting the diagnostic accuracy of either eNose or GC–MS for the discrimination of urine samples from people with PCa and healthy controls. As no randomised studies have been performed in this area, non‐randomised studies including cohort and case–control studies were included. Targeted and untargeted metabolomic studies were eligible for inclusion. Any means of gaining a diagnosis of PCa was accepted as a reference standard (for example, biopsy, incidental findings on TURP histology, or clinical diagnosis using imaging findings and elevated PSA).

Exclusion criteria included conference proceedings, due to a lack of quality assurance in the form of peer review, and studies not reporting either a 2 × 2 contingency table or sensitivity, specificity and size of each group. Any targeted metabolomic studies specifically investigating sarcosine, which was explored as a potential diagnostic biomarker for PCa in the early 2010s but has since been refuted in multiple high‐quality studies were also excluded.

## 3 EVIDENCE SYNTHESIS

Data were extracted separately for GC–MS and eNose‐based studies. The following characteristics of each study were recorded in the data extraction tool: publication year, lead author, study design, number of participants (cancer/total), control group characteristics, analytical platform used (GC–MS or eNose), sample processing, statistical techniques, sensitivity and specificity (with 95% confidence intervals where reported). For studies using GC–MS the names of compounds included in the model were recorded. In cases of missing data, the corresponding authors were contacted by email and requested to provide information where available. Where authors did not respond or could not provide further information, the number of true positives, true negatives, false positives and false negatives were calculated from reported sensitivity/specificity and numbers of PCa cases and healthy controls in each study. 95% confidence intervals were calculated if not otherwise reported. Wherever inferred values have been calculated, these were recorded in the data extraction tool.

The risk of bias and applicability of each study were assessed using the Quality Assessment of Diagnostic Accuracy Studies 2 (QUADAS 2) tool. The quality of reporting was assessed using the Standards for Reporting Diagnostic Accuracy Studies (STARD) checklist.

The sensitivity and specificity of each study for the diagnosis of PCa were represented in forest plots. Based on the authors' experience of metabolomic studies and a preliminary exploratory review of the literature a meta‐analysis of diagnostic accuracy statistics was not performed (by advanced decision) due to a high degree of methodological heterogeneity, including different materials for eNose devices and different groups of compounds used for model development. A narrative review describing compounds identified using GC–MS was constructed. Finally, recommendations for improving future metabolomic studies for the discrimination of PCa from healthy controls were proposed.

## 4 RESULTS

### 4.1 Study selection

**FIGURE 1:**
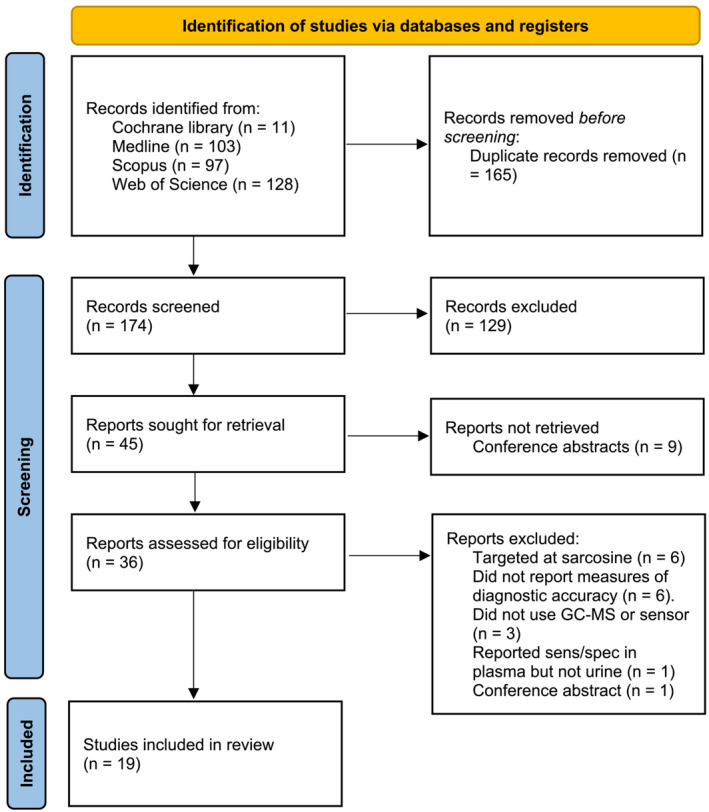
PRISMA flow chart detailing the process for identifying eligible studies.

A total of 339 records were identified. After deduplication, 174 abstracts were screened for relevance to the research question. Forty‐five of these were sought for retrieval and nine were excluded as they were conference proceedings. Of the remaining 36 that were assessed for eligibility, 19 were included in the final review (see Figure 1). Reasons for excluding other studies included studies targeting sarcosine (n = 6), no measure of diagnostic accuracy or means to calculate them (n = 6), neither GC–MS or eNose used (n = 3), results for urine not reported (n = 1) and conference proceeding (n = 1). Figure S3 summarises a list of the excluded studies.

### 4.2 Diagnostic Capability of eNose

Of the studies utilising eNose, the first was completed by Asimakopoulos et al.^11^ in 2014. This used an array of eight tetraphenylporphyrin complexes of copper, cobalt, zinc, manganese, iron, tin, ruthenium and chromium and reported a sensitivity of 71.4% and specificity of 92.6% in a sample of 41 patients, including 14 with known PCa.

Three studies from the same laboratory utilised metal oxide sensors.^12^, ^13^, ^14^ Bax et al. produced a proof‐of‐concept study demonstrating that inkjet printers could be used to manufacture reliable zinc oxide sensors,^12^ following which Capelli et al. and Taverna et al. utilised an array of six sensors composed of zinc, titanium and tin oxides produced using the inkjet methodology.^13^, ^14^ Sensitivity and specificity for this technology has been consistently high at 79.5–85.2% and 79.1–87%, respectively.

**FIGURE 2:**
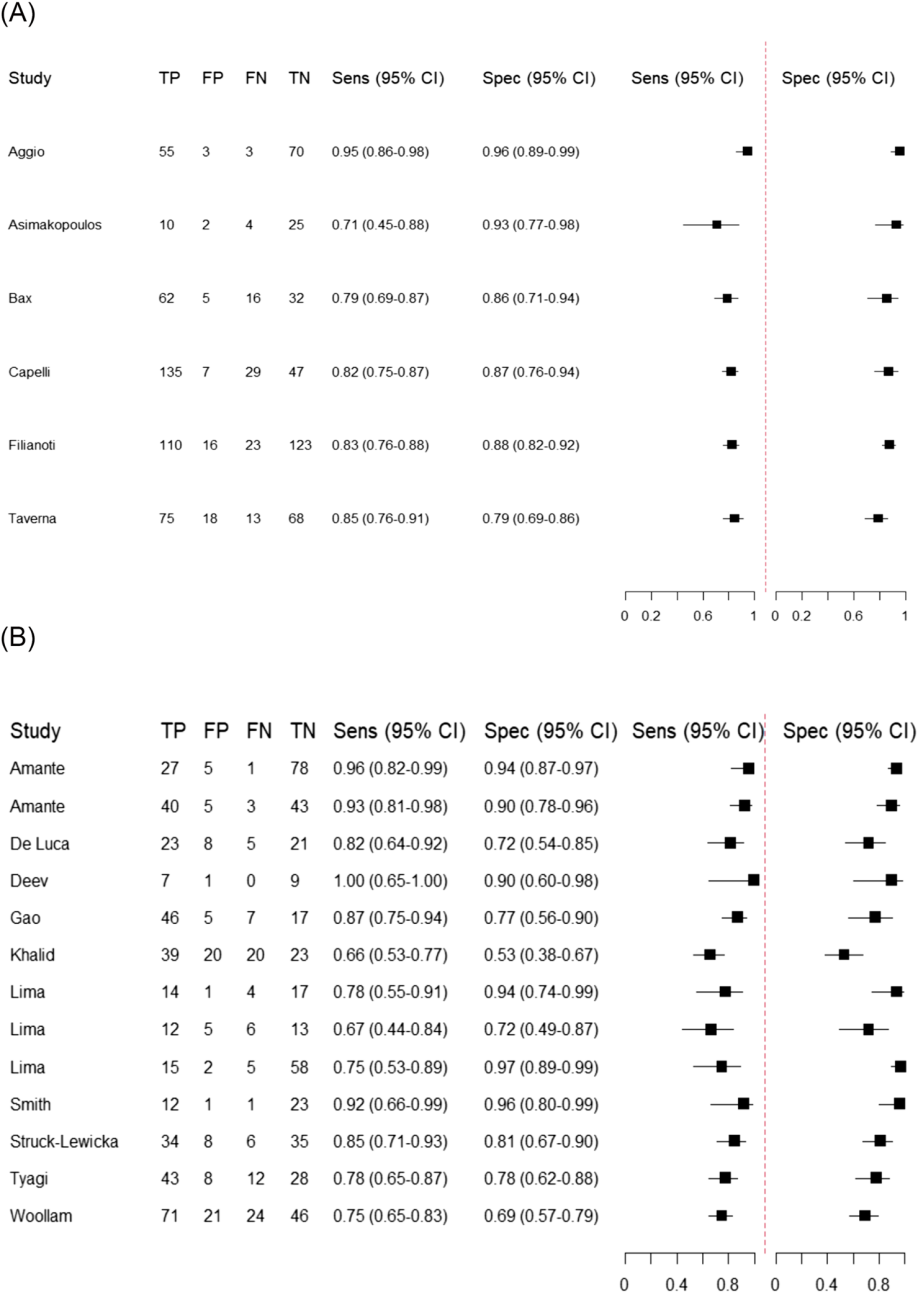
Forest plots of sensitivity and specificity for (a) eNoses and (b) GC–MS in the discrimination of urine headspace gases from patients with PCa and cancer‐free controls.

Aggio et al. utilised a novel technique combining gas chromatography with a zinc oxide/tin oxide sensor, achieving a sensitivity and specificity of 93.1–94.8% and 95.9%, respectively, depending on the statistical techniques used to build the models.^15^ This was the only eNose‐based study using alkali to adjust urinary pH. Filianoti et al. reported the only use of a commercially available polymer sensor‐based eNose as opposed to an in‐house prototype system.^16^ They achieved similar results to other eNose‐based studies, demonstrating sensitivity and specificity of 83.1% and 87.6%, respectively, in a sample of 133 PCa cases and 139 controls, the largest study involving eNose technology. Further details of each study are summarised in Table 1 and a summary of sensitivities and specificities for each study is shown in Figure 2.

**TABLE 1:** Study characteristics for studies utilising eNoses.

Author	Year	Design	Participants (cancer, controls)	Control group characteristics	Technique	Sample processing	Statistical analysis for model building
Aggio^15^	2016	Cohort	58 cancer, 73 controls	Men under investigation for LUTS, abnormal DRE or raised PSA	GC‐MOS	0.75 ml urine, 0.75 ml 1 M NaOH	Linear discriminant analysis
Asimakopoulos^11^	2014	Cohort	14 cancer, 27 controls	Caucasian men referred for prostate biopsy with negative result	MPS	None	PLS‐DA
Bax^12^	2021	Case–control	78 cancer, 37 controls	Non‐age‐matched men with no history of prostate cancer	MOS	None	Random forest
Capelli^13^	2021	Case–control	132 cancer, 60 controls	Healthy females, young males, males over 60 with low PSA	MOS	None	Random forest
Filianoti^16^	2022	Case–control	133 cancer, 139 controls	Patients with no known prostate cancer (no previous biopsy)	Organic polymer sensors	None	PCA
Taverna^14^	2022	Case–control	88 cancer, 86 controls	Healthy females, young males, males over 45 with low PSA	MOS	None	Not reported

LUTS: lower urinary tract symptoms; DRE: digital rectal examination; PSA: prostate‐specific antigen; TURP: transurethral resection of the prostate; GC: gas chromatography; MOS: metal oxide sensor; MPS: metalloporphyrin sensor; PLS‐DA: partial least squares discriminant analysis; PCA: principal component analysis.

### 4.3 Diagnostic Capability of GC–MS

Of studies using GC–MS for the detection of PCa in urine headspace, Tyagi et al^17^ were the only authors to use unprocessed urine. They applied two different technologies, GC‐time of flight MS (GC‐TOF‐MS) and GC‐ion mobility spectrometry (GC‐IMS), to differentiate prostate cancers from healthy individuals. GC‐TOF‐MS was found to have better discriminatory potential with a sensitivity and specificity of 78% and 88%, respectively. They also assessed if this technology could differentiate between bladder and prostate cancers, showing a specificity of 90%.

Three studies alkalinised urine with NaOH prior to GC–MS analysis.^18^, ^19^, ^20^ In these, Smith et al. reported high sensitivity and specificity (92.3% and 96.3%, respectively) in a small pilot study of 37 patients.^19^ However, this finding was not replicated by Khalid et al. in the first prospective cohort study of GC–MS for PCa discrimination, with sensitivities of 66% and 87% and specificities of 53% and 31% using linear discriminant analysis and random forest classification, respectively.^18^

More recently, Woollam et al. conducted a cohort study, this time adding NaCl to alkalinised urine and using GC‐quantitative TOF‐MS, achieving a sensitivity and specificity of 75% and 69%, respectively.^20^ This is the only study so far to utilise GC–MS for risk stratification of PCa, reporting a panel of seven VOCs that could discriminate high‐grade PCa (ISUP grade group 3–5) from low‐grade (ISUP grade group 1–2) with a sensitivity and specificity of 78% and 85%, respectively.

Lima et al. conducted three studies using GC–MS for PCa diagnosis.^21^, ^22^, ^23^ They constructed a model using salted urine which could differentiate PCa from healthy controls with a sensitivity and specificity of 78 and 94%, respectively(21). To investigate the hypothesis that specificity may be lost when compared to other urinary tract cancers (for example, due to metabolic aberrations common in cancers such as altered glucose metabolism), they tested and built upon it by using a control group containing patients with known bladder or renal cancer, achieving a sensitivity and specificity of 76% and 97%, respectively.^23^ The third study used GC–MS and proton nuclear magnetic resonance (^1^H NMR) spectroscopy, and demonstrated that ^1^H NMR could identify a smaller range of VOCs than GC–MS.^22^ GC–MS identified 151 compounds, whilst ^1^H NMR identified 52. Ten compounds were identified using both modalities, suggesting that ^1^H NMR may be useful in extending the range of compounds for metabolomic studies. In external validation sets, GC–MS achieved higher sensitivity (89% vs 67%) but slightly lower specificity (83% vs 89%) than ^1^H NMR.

Gao et al. acidified urine with HCl prior to analysis in a prospective cohort study and demonstrated a sensitivity and specificity of 87% and 77%, respectively.^24^ Amante et al. added either acid or alkali to centrifuged urine to achieve a target pH of 6.8–7.4. They performed extensive pre‐processing of the pH‐standardised samples including enzymatic hydrolysis using β‐glucurinidase, liquid–liquid extraction, drying under nitrogen steam and addition of solvents before direct injection into the GC–MS. GC–MS analysis using this method resulted in a sensitivity and specificity of 92.5% and 88.7%, respectively.^25^

Two studies reported targeted metabolomic analysis using urinary steroid profiles by GC–MS with the same methodology. Amante et al reported a high sensitivity and specificity of 96% and 94%, respectively.^26^ The model developed by De Luca et al. showed a lower sensitivity and specificity of urinary steroid profiles of 82% and 73%.^27^ A summary of study characteristics is shown in Table 2, and sensitivities and specificities for all these studies can be found in Figure 2. Table 3 summarises the specific VOCs used in each study for model building.

**TABLE 2:** Summary of characteristics for studies using GC–MS for the analysis of urinary headspace VOCs for the discrimination of patients with PCa from healthy controls. GC: gas chromatography; MS: mass spectrometry; PLS‐DA: partial least squares discriminant analysis; LDA: linear discriminant analysis; IMS: ion mobility spectrometry; (Q)TOFMS: (quantitative) time of flight mass spectrometry.

Author	Year	Study design	Participants	Control group characteristics	Technique	Sample processing	Statistical analysis	Compounds targeted
Amante^26^	2018	Case–control	29 cancer, 83 controls	Healthy caucasian men aged >60	GC–MS	6 ml urine, pH adjusted to 7.5 with buffer and NaOH, centrifuged, dried and resuspended in MSTFA/NH4I/dithioerythritol, 1 μl injected into GC–MS	PLS‐DA	Urinary steroids
Amante^25^	2019	Case–control	43 cancer, 48 controls	BPH with a PSA < 4 ng/ml or with a PSA or negative biopsy	GC–MS	Addition of 1 M NaOH or HCl to achieve pH > 10 or <1, dried, dissolved in 50 μl TBME	PLS‐DA	Untargeted
De Luca^27^	2021	Case–control	144 cancer, 139 controls	Previous negative mpMRI and biopsy	GC–MS	6 ml urine, pH adjusted to 7.5 with buffer and NaOH, centrifuged, dried and resuspended in MSTFA/NH4I/dithioerythritol, 1 μl injected into GC–MS	PLS‐DA	Urinary steroids
Deev^28^	2020	Case–control	20 cancer, 30 controls	Men with no known urinary tract pathology (no previous biopsy)	GC–MS	3 ml urine with 0.3 g NaCl added	K‐nearest neighbour and PLS‐DA	Untargeted
Gao^24^	2019	Cohort	55 cancer, 53 controls	Men referred for prostate biopsy with negative result	GC–MS	1 ml urine, 19 ml deionised water, 600 μl 2 M HCl and 300 μl Mirex (internal standard)	Regularised logistic regression	Untargeted
Khalid^18^	2015	Cohort	59 cancer, 43 controls	Men referred for prostate biopsy with negative result	GC–MS	0.75 ml urine, 0.75 ml 1 M NaOH	LDA and random forest	Untargeted
Lima^21^	2019	Case–control	58 cancer, 60 controls	Men aged 56–66, not reported further	GC–MS	1 ml urine, 0.27 g NaCl	PLS‐DA	Untargeted
Lima^22^	2020	Case–control	59 cancer, 60 controls	Men, mean age 59, not reported further	GC–MS	200 μl urine, 20 μl urease suspension, supernatant evaporated to dryness, 100 μl toluene added to the residue	PLS‐DA	Untargeted
Lima^23^	2020	Case–control	20 cancer, 60 controls	20 bladder cancer, 20 renal cancer, 20 no cancer	GC–MS	1 ml urine, 0.27 g NaCl	PLS‐DA	Untargeted
Smith^19^	2010	Case–control	13 cancer, 24 controls	Asymptomatic men with no known cancer	GC–MS	0.75 ml urine, 0.75 ml 1 M NaOH	Simple Matching Coefficient, Jaccard, Tanimoto, Russell and Rao	Untargeted
Struck‐Lewicka^29^	2020	Case–control	40 cancer, 43 controls	Age‐ and BMI‐matched men with no known cancer	GC–MS	200 μl urine, 50 μl urease suspension, 800 μl methanol, 10 μl pentadecanoic acid added. Supernatant evaporated to dryness, 30 μl methoxyamine in pyridine added to the residue. Heated, 30 μl BSTFA, 1% TMCS, 70 μl heptane added.	OPLS‐DA	Untargeted
Tyagi^17^	2021	Case–control	55 cancer, 36 controls	Males and female with no known cancer	GC‐TOFMS	None	Random forest	Untargeted
Woollam^20^	2023	Cohort	95 cancer, 67 controls	Men referred for prostate biopsy with negative result	GC‐QTOFMS	2.5 ml urine, 0.9 g NaCl, corrected to pH 6.5–7 with 1 M NaOH	Linear discriminant analysis	Untargeted

**TABLE 3:** Summary of VOCs included in statistical models in studies using GC–MS for the detection of PCa from urine headspace VOCs. NR: not reported.

Author	Total VOCs	Upregulated compounds in PCa	Downregulated compounds in PCa	Not mentioned whether up or down
Amante^26^	8	Testosterone, androsterone, 5β‐androstan‐3α,17β‐diol, 5α‐androstan‐3α,17β‐diol, 4‐hydroxytestosterone, dehydroepiandrosterone, 7β‐hydroxydehydroepiandrosterone, etiocholanolone		
Amante^25^	32	5‐hydroxyindoleacetic acid, androsterone, 16‐hydroxydehydroisoandrosterone, vanillyl alcohol, 4 unknown compounds	Enterodiol, 5β‐pregnanediol, pregnanetriol, 15 unknown compounds	Epiandrosterone, 5 unknown compounds
De Luca^27^	3	7β‐hydroxydehydroepiandrosterone, testosterone, etiocholanolone	Nil	
Deev^28^	5			(5β)‐Androst‐9(11)‐ene‐3,17‐dione, Estr‐4‐ene‐3,17‐diol, 3‐Ethyl‐3‐hydroxyandrostan‐17‐one, Androst‐5‐ene‐3,17‐diol, Androst‐2‐en‐17‐one
Gao^24^	11	1,1,3,3,5,5,7,7,9,9‐decamethyl‐pentasiloxane, 1,1,1,5,5,5‐hexamethyl‐3,3‐bis[(trimethylsilyl)oxy]‐Trisiloxane, Phthalic acid, bis(7‐methyloctyl) ester, 4‐Nitro‐4′‐chlorodiphenylsulfoxide, 1‐Propylpentachlorotriphosphazene, 2,6‐di‐t‐butyl‐4‐hydroxymethylene‐2,3,5,6‐detetrahydrocyclohexanone	4‐(3,4‐dihydro‐2,2,4‐trimethyl‐2H‐1‐benzopyran‐4‐yl)‐phenol, Estradiol, Ethyl α‐hydroxymyristate trisiloxane, 1‐(2,4‐Dimethylphenyl)‐3‐(tetrahydrofuryl‐2)propane, 2‐amino‐Imidazole‐5‐carboxylic acid	
Khalid^18^	4	Pentanal	2,6‐dimethyl‐7‐octen‐2‐ol, 3‐octanone, and 2‐octanone	
Lima^21^	6	2,5‐dimethylbenzaldehyde, 3‐phenylpropionaldehyde	hexanal, 4‐methylhexan‐3‐one, dihydroedulan IA, methylglyoxal	
Lima^22^	NR	NR	NR	
Lima^23^	10	2,5‐dimethylbenzaldehyde, 3‐phenylpropionaldehyde, ethylbenzene, heptan‐2‐one, methyl benzoate, 3‐methyl‐benzaldehyde	hexanal, 4‐methylhexan‐3‐one, dihydroedulan IA, methylglyoxal	
Smith^19^	21	Butyrolactone, methyl vinyl ketone, methylamine, N‐ethylformamide, acetonitrile dimethylamino, pyridine, N‐methylformamide, acetaldehyde, acetamide, 1‐methylpiperidine, 1‐piperidine acetonitrile, dimethylamine, pyrrole, methacrolein, N,N‐dimethylformamide, 3‐methylpyridine, 2‐methyl‐1H‐pyrrole, 2‐octanone, 1‐ethyl‐1H‐pyrrole, 2‐n‐butylacrolein, methyl propyl disulfide		
Struck‐Lewicka^29^	11	Nil	Hexose, Aconitic acid, lactose, ribonic acid, erythritol, inositol, cellobiose, phenylacetic acid, sucrose, hippuric acid, galactinol	
Tyagi^17^	7			Toluene, phenol, acetic acid, 2‐ethylhexan‐1‐ol, dimethyl disulfide, 2‐methylcyclopentane, pyrrole
Woollam^20^	6	NR	NR	NR

### 4.4 Risk of bias and applicability issues

For eNose‐based studies, a low risk of bias was observed in the utilisation of reference standards. All studies either used patients that were undergoing prostate biopsy, the gold standard for diagnosis of PCa, or already had a diagnosis of PCa for which they were being treated. Only one study specified whether transrectal or transperineal biopsy was performed,^11^ however both routes are thought to be equally effective in identifying the presence of prostate cancer according to European guidelines.^3^ Five out of seven studies were found to have a high risk of bias in both patient selection and flow and timing aspects of the QUADAS 2 checklist.^12^, ^13^, ^14^, ^16^ These studies recruited patients with known PCa after their biopsy results were already known. Furthermore, the time interval between the index test and the reference standard meant there was a risk that patients may have had more advanced disease than described in the results if not treated, or conversely could have already undergone treatment resulting in less or no detectable disease.

**FIGURE 3:**
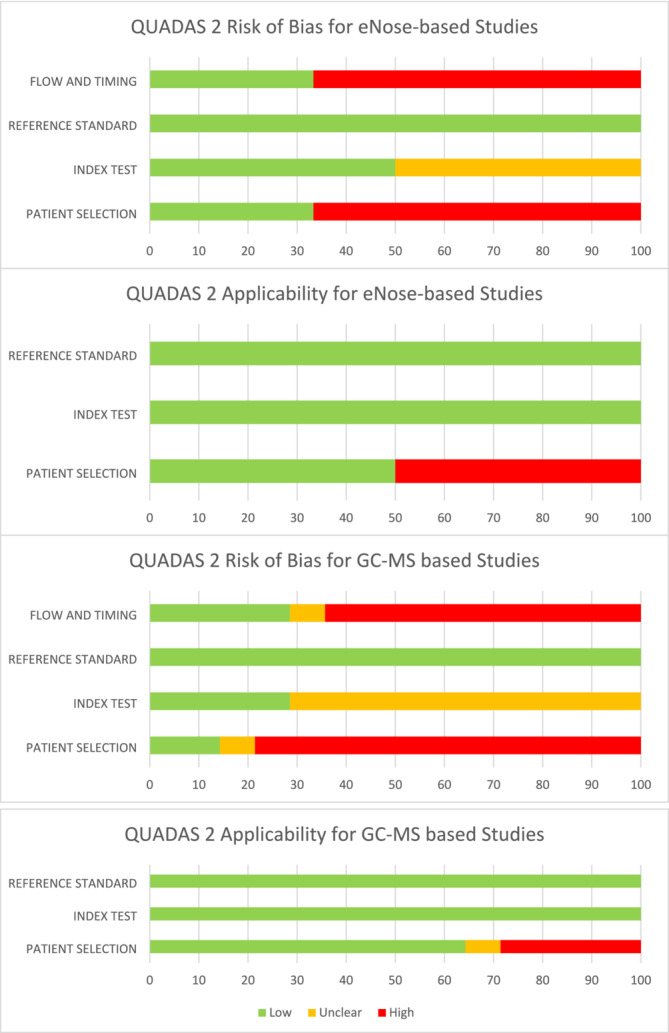
QUADAS 2 results for eNose‐ and GC–MS‐based studies.

Applicability of studies was generally found to be good, however, three were found to have applicability concerns due to inappropriate control group members, including a significantly younger age or the inclusion of female patients.^12^, ^13^, ^14^ See Figure 3 for a summary of QUADAS 2 results for eNose‐based studies.

GC–MS‐based studies had a similar pattern of results in the QUADAS 2 checklist (see Figure 3). Most studies were found to have high risks of bias in patient selection and flow and timing for similar reasons to the eNose‐based studies. Applicability was generally good, with some studies again scoring high risk for applicability concerns due to inappropriate control groups.^17^, ^23^, ^25^

Using the STARD checklist, reporting standards in the eNose‐based studies were generally higher than those in the GC–MS‐based studies. The majority of studies scored highly in the title, abstract and introduction sections, giving clear clinical and biological background to justify the need for new biomarkers for PCa and the rationale for investigating VOCs. The index standard was usually described in enough detail to replicate the methods; however, descriptions of the reference standard were usually limited to a general statement about having a biopsy in the past. Justification of sample size and handling of indeterminate tests for both index test and reference standard were not reported across all studies. eNose‐based studies generally reported more information about participants including the range of severity of disease in those with PCa and the time interval between the index test and reference standard. In the results sections, although all studies reported sensitivity and specificity, five eNose‐based studies and two GC–MS‐based studies reported 95% confidence intervals for these statistics. Full results of the STARD checklist can be found in Figure S2.

One area of concern for the majority of papers was a lack of reporting of clinical and demographic characteristics. Most studies reported some detail of the age ranges of their participants, but it should be noted that several studies had large differences between the age of their cases and controls,^23^, ^30^, ^31^ and others did not report cases and controls separately.^11^, ^20^, ^26^ Important risk factors for PCa such as ethnicity^11^, ^25^, ^26^ and family history^31^, ^32^ were rarely reported. Six studies did not report PSA results for participants,^12^, ^15^, ^19^, ^20^, ^26^, ^29^ while a further five did not report PSA results in their control groups.^21^, ^22^, ^23^, ^30^, ^33^ Only two studies mentioned whether patients underwent MRI scanning prior to biopsy. De Luca et al. was the only study to specify that all patients underwent MRI but did not report any of these results,^27^ whilst Filianoti et al. reported radiological stage for their cases but did not report whether controls underwent MRI.^34^ For full clinical and demographic details please see Table S1.

## 5 DISCUSSION

This systematic review has demonstrated that both eNose and GC–MS have reasonable sensitivity for the detection of PCa, with values ranging from 0.71 to 0.95 and 0.66 to 1.00, respectively, for each. Specificities for eNose and GC–MS ranged from 0.79 to 0.96 and 0.53 to 0.97, respectively. Confidence intervals for eNose studies were generally very narrow and consistent in comparison to corresponding figures for GC–MS studies. In particular, Aggio et al. reported a very high accuracy for their novel GC‐sensor device.^15^ eNoses are readily available to purchase for non‐medical purposes, such as environmental air quality monitoring, so their use in clinical laboratories ‐ if regulatory approval was achieved ‐ could be rolled out much more easily than GC–MS, which is both cumbersome and requires specialist training.

A recent meta‐analysis of the use of PSA in 20 studies, of which 19 were based in urology outpatient clinics, demonstrated a high sensitivity of 0.93 (95% CI 0.88–0.96) but a lower specificity of 0.20 (95% CI 0.12–0.33).^4^ Whilst these results cannot be directly compared, they have at least been conducted in similar populations with similar reference standards. A Cochrane review found similar sensitivity (0.91 [95% CI 0.83–0.95]) and higher specificity (0.37 [95% CI 0.29–0.46]) for MRI,^35^ the current tool recommended for selecting out patients unlikely to have PCa. While there are doubtless advantages to performing an MRI for lesion localisation and staging purposes, urinary VOC analysis may have a role as a low‐cost, non‐invasive tool to improve patient selection either prior to MRI or in men with PIRADs 3 lesions prior to biopsy.

A 15‐year follow‐up from the ProtecT trial demonstrates low disease‐specific mortality in localised PCa, with authors citing a need for improved protocols for ruling out indolent disease.^36^ So far, only one study has explored whether PCa can be risk‐stratified based on urinary VOC profiles.^20^ It suggested a potential role for urinary VOC analysis in selecting patients with Grade Group 3–5 PCa who are likely to benefit from radical treatment. The VOCs identified in this study would require external validation prior to being utilised in clinical practice and it is clear more work is needed to define and investigate VOCs as biomarkers for csPCa.

Analysis using the QUADAS 2 checklist revealed some concerns regarding the risk of bias in most studies. These primarily concerned patient flow through the studies. Authors often implied that recruited patients had previously been diagnosed with PCa, i.e. sampling took place after their diagnostic biopsy. This retrospective recruitment strategy increases the risk of recruitment bias. It is unknown whether the biopsy procedure itself alters the VOC profile of urine either through the inflammatory response to tissue trauma or through the introduction of bacteria into the prostate gland. Furthermore, the time interval between the diagnosis of PCa and the reference test was often not described, meaning patients with PCa may have actually had more advanced disease than described in the results, limiting the applicability of the index tests in cases of low‐grade, localised disease which is where the greatest diagnostic uncertainty of the current pathway lies. Similarly, the effects of treatment on VOC profiles are also unknown.

QUADAS 2 results indicated that authors generally reported the methodology for the index test in sufficient detail for it to be replicated easily by other laboratories. However, the reference standard was not always described, or multiple reference standards were used for different patients and some studies recruited control groups that had no reference standard at all. Furthermore, reporting of demographic and clinical details is often poor, with little information about PSA and staging of tumours. This makes it difficult to know whether the study populations are generalisable to the wider population and whether recruitment bias has influenced the reported outcomes. Where factors are reported, there are often significant differences in demographic factors including age.

The focus on the instrumentation and lab techniques used reflects the fact that metabolomics is an emerging science: most studies included in this review are small‐scale pilot studies exploring an, as yet, unstandardised methodology with novel techniques and devices. Based on the early positive results presented in this review, there is a need for larger‐scale, well‐designed and properly funded studies to validate these early findings. Recent well‐powered, prospective studies have so far supported early findings suggesting a role for VOC analysis in the detection of PCa.^15^, ^20^

As expected, there is methodological heterogeneity between studies. GC–MS studies used varying volumes of urine and processed them with different types and concentrations of acids, alkalis and solvents. These factors affect the VOC profile of the urinary headspace and may explain why there is little crossover between compounds used in statistical models.^10^ eNose studies from different laboratories used different devices, as there is no industry standard for this technology. For this reason, no meta‐analysis was performed as any result would be invalid.

This review has limitations of its own. Firstly, some of the 95% confidence intervals had to be calculated from the sensitivity, specificity and sample sizes presented in reviews. Whilst authors were contacted in all cases of missing data, not all responded and therefore this was the only means of presenting this metric. Secondly, no consideration has been given to other metabolomic techniques, such as liquid chromatography‐mass spectrometry or nuclear magnetic resonance spectroscopy. This could be a potential focus for a future review.

There are two areas where this review has varied from the original protocol. First, the protocol stated we would only include studies using trans‐rectal or trans‐perineal prostate biopsy as a reference standard. Due to poor quality reporting of reference standards across studies and lack of reporting of biopsies in control groups, it was decided to include all patients with a clinical diagnosis of prostate cancer even if the modality of diagnosis was not specifically reported. If we had adhered to the original protocol, only one eNose study and four GC–MS studies would have been included in the review. It is therefore important to interpret these results in the context of the available clinical data. Secondly, the protocol stated that disagreement in a decision on whether to include a study would be settled by discussion with the supervising author. It became apparent that the supervising author was in fact a named author on several of the included studies. Therefore, to minimise bias in the study selection process any disagreements were resolved by consensus between the two screening authors with no involvement from the supervising author.

## 6 CONCLUSIONS

This systematic review has shown GC–MS and eNose to be reasonably sensitive for detecting prostate cancer in urine. Both technologies appear more specific than both PSA and mpMRI, so may have a role in reducing the harm caused by unnecessary biopsies. It should be noted that these studies are generally small, represent a range of different technologies and have not yet been fully validated. Limited data are available to determine the utility of urinary VOC analysis on the discrimination of csPCa from indolent disease. Future work should focus on conducting larger, prospective studies to build on this promising early work.

## AUTHOR CONTRIBUTIONS

**Jonathon Dawson:** Conceptualisation; data extraction; data analysis; writing—initial draft. **Kraig Green:** Data extraction and manuscript revision. **Henry Lazarowicz:** Manuscript revision. **Phil Cornford:** Manuscript revision. **Chris Probert:** Manuscript revision; project supervision. All authors discussed the results and contributed to the final manuscript.

## CONFLICT OF INTEREST STATEMENT

We wish to confirm that there are no known conflicts of interest associated with this publication and there has been no significant financial support for this work that could have influenced its outcome. We confirm that the manuscript has been read and approved by all named authors and that there are no other persons who satisfied the criteria for authorship but are not listed. We further confirm that the order of authors listed in the manuscript has been approved by all of us. We confirm that we have given due consideration to the protection of intellectual property associated with this work and that there are no impediments to publication, including the timing of publication, with respect to intellectual property. In so doing we confirm that we have followed the regulations of our institutions concerning intellectual property.

## Supporting information


**Figure S1:** Search Strategy.
**Table S1:** Clinical Characteristics of Included Patients.
**Figure S2:** STARD Results.
**Figure S3:** Excluded Studies.
