# Supplementary material for: Analysis of urinary volatile organic compounds for prostate cancer diagnosis: A systematic review
Source: BJUI Compass. 2024 Aug 6;5(9):822–33. doi: 10.1002/bco2.423 (PMC11420098; doi:10.1002/bco2.423)
Supplement: Supplementary file 1 — Figure S1: Search Strategy. Table S1: Clinical Characteristics of Included Patients. Figure S2: STARD Results. Figure S3: Excluded Studies. [file BCO2-5-822-s001.docx]

Supplementary Figure 1: Search Strategy

1 Volatile Organic Compounds

2 VOC*.ti,ab,kw.

3 Volatile organic compound*.ti,ab,kw.

4 GC-MS.ti,ab,kw.

5 Gas chromatography mass spectrometry.ti,ab,kw.

6 Odour reader.ti,ab,kw.

7 eNose.ti,ab,kw.

8 GC-sensor.ti,ab,kw.

9 FIAMS.ti,ab,kw.

10 Metabolomic*.ti,ab,kw.

11 (prostat* adj3 cancer).ti,ab,kw.

12 (prostat* adj3 carcinoma).ti,ab,kw.

13 (prostat* adj3 malignancy).ti,ab,kw.

14 (prostat* adj3 adenocarcinoma).ti,ab,kw.

15 (prostat* adj3 neoplasm*).ti,ab,kw.

16 Prostatic Neoplasms/

17 Urin*.ti,ab,kw.

18 1 or 2 or 3 or 4 or 5 or 6 or 7 or 8 or 9 or 10

19 11 or 12 or 13 or 14 or 15 or 16

20 17 and 18 and 19

Supplementary Table 1: Clinical Characteristics of Included Patients

| **Study** | **Year** | **Country** | **Setting** | **Age (years)** | **Ethnicity** | **Family history** | **PSA (ng/ml)** | **% receiving MRI** | **Clinical stage** | **Grade (cases only)** | **Reference test** |
| --- | --- | --- | --- | --- | --- | --- | --- | --- | --- | --- | --- |
| **GC-MS** | | | | | | | | | | | |
| Amante | 2018 | Italy | Single centre urology outpatient department | All >60, not reported further | Caucasian | NR | NR | NR | NR | NR | Prostate biopsy (type not reported) |
| Amante | 2019 | Italy | Single centre urology outpatient department | Cases: mean 70 (SD 10)  Controls: 70 (SD 8) | Caucasian | NR | Cases 11 (9.5)  Controls: 3.8 (2.3) | NR | NR | Gleason 6: 15 (35%)  Gleason 7: 21 (49%)  Gleason >7: 7 (16%) | Prostate biopsy (type not reported) |
| De Luca | 2021 | Italy | Single centre urology outpatient department | Cases: median 68 (IQR 63-74)  Controls median 67.8 (IQR 62-73) | NR | NR | Cases: median 6.8 (IQR 4.8-14.4)  Controls: median 2.6 (1.0-4.7) | 100% | Cases: 4.9% <cT2a  Controls: 0.7% <cT2a | NR | TRUS biopsy |
| Deev | 2020 | Russia | Single centre urology outpatient department | NR. Control group age matched to cases | NR | NR | Cases: range 2.5-298  Controls NR | NR | NR | NR | Prostate biopsy (type not reported) |
| Gao | 2019 | USA | Single centre internal medicine outpatient department | NR | NR | NR | Cases: median 3.75 (IQR 0.08-75)  Controls: median 5.21 (1.8-20) | NR | NR | Gleason 6: 31 (58%)  Gleason 7: 16 (30%)  Gleason 8: 3 (6%)  Gleason 9: 3 (6%) | TRUS biopsy |
| Khalid | 2015 | UK | Single centre urology outpatient department | Cases: median 69 (range 50-88)  Controls: median 63 (range 41-81) | NR | NR | Cases: median 10.2 (range 3.4-647)  Controls: median 6.2 (0.8-30) | NR | NR | NR | TRUS biopsy |
| Lima | 2019 | Portugal | Single centre oncology outpatient department | Cases: mean 61.8 (SD 5.2) Controls: mean 63.4 (SD 5.3) | NR | NR | Cases:  <4 1 (5.6%)  4-10 13 (72.2%)  >10 4 (22.2%)  Controls: NR | NR | Cases:  I 3 (16.7%)  II 2 (11.1%)  IIA 4 (22.2%)  IIB 2 (11.1%)  III 5 (27.8%)  IV 2 (11.1%) | Gleason 6: 3 (16.7%)  Gleason 7 12 (66.7%)  Gleason ≥8 3 (16.7%) | NR |
| Lima | 2020 | Portugal | Single centre oncology outpatient department | Cases: mean 61.8 (SD 5.9) Controls 60.2 (SD 3.35) | NR | NR | Cases:  <4 1 (5.6%)  4-10 14 (77.8%)  >10 3 (16.7%)  Controls NR | NR | Cases:  I 2 (11.1%)  II 11 (61.1%)  III 4 (22.2%)  IV 1 (5.6%) | Gleason 6: 2 (11.1%)  Gleason 7: 14 (77.8%)  Gleason 8 1 (5.6%)  Gleason 9 1 (5.6%) | NR |
| Lima | 2020 | Portugal | Single centre oncology outpatient department | Cases: mean 67 (SD 8.1) Controls 58 (SD 2.8) | NR | NR | Cases:  <4 1 (5%)  4-10 7 (35%)  >10 4 (20%)  unavailable 8 (40%)  Controls NR | NR | Cases:  I 7 (35%)  II 3 (15%)  III 2 (10%)  IV 6 (30%)  not available 2 (10%) | Gleason 6: 7 (35%)  Gleason 7: 9 (45%)  Gleason ≥8 3 (15%)  Unavailable 1 (5%) | NR |
| Smith | 2010 | UK | NR | Cases: mean 75.2 (SD 6.9) Controls: mean 62.9 (SD 5.5) | NR | NR | NR | NR | NR | NR | NR |
| Struck-Lewicka | 2020 | Poland | Single centre urology and family medicine outpatient departments | Cases: mean 65.2 (SD 6.3)  Controls NR but age matched | NR | NR | NR | NR | NR | Gleason ≤6: 7 (17.5%)  Gleason 7: 19 (47.5%)  Gleason ≥8: 4 (10%)  Unknown 10 (25%) | Prostate biopsy (type not reported) |
| Tyagi | 2021 | UK | Single centre, not otherwise specified | Cases: mean 71.9  Controls: mean 62.5 | NR | NR | Cases: mean 20.6 (range 3.6–153.90)  Controls: NR | NR | NR | Gleason 6: 11 (20%)  Gleason 7: 24 (60%)  Gleason ≥8: 20 (40%) | NR |
| Woollam | 2023 | USA | Single centre urology outpatient department and pre-operative assessment | Range for all participants: 40-85 | NR | NR | NR | NR | NR | ISUP 1: 38 (40%)  ISUP 2: 30 (32%)  ISUP 3: 8 (8%)  ISUP 4: 5 (5%)  ISUP 5: 14 (15%) | Prostate biopsy (type not reported) |
| **eNose** | | | | | | | | | | | |
| Aggio | 2016 | UK | Single centre prostate biopsy and flexible cystoscopy clinics | Cases: median 69 (range 50-88)  Controls: median 64 (range 29-86) | NR | NR | NR | NR | NR | Gleason ≤6: 34 (59%)  Gleason 7: 20 (34%)  Gleason ≥8: 4 (7%) | Prostate biopsy (type not reported) |
| Asimakopoulos | 2014 | Italy | Single centre prostate biopsy clinic | Range for all participants: 54-77 | Caucasian | NR | Range for all participants: 1.95-14.02 | NR | NR | Gleason 6: 12 (86%)  Gleason 7: 2 (14%) | Transperineal biopsy |
| Bax | 2021 | Italy | Single centre, not otherwise specified | NR | NR | NR | NR | NR | NR | NR | Prostate biopsy or incidental cancer on TURP |
| Capelli | 2021 | Italy | Single centre, not otherwise specified | Cases: range 46-84  Controls: range 10-75 | NR | Controls: no family history  Cases: NR | Cases: range 0.6-78  Controls: <5.5 | NR | Cases: all cT1c-cT3 | Gleason 6-7: 77 (58%)  Gleason ≥8: 48 (36%)  NR: 7 (5%) | NR |
| Filianoti | 2022 | Italy | Single centre, not otherwise specified | Cases: mean 67.37 (SD 6.1)  Controls: mean 65.97 (SD 12.99) | NR | NR | Cases: mean 12.65 (SD 37.05)  Controls: mean 3.34 (SD 4.64) | Cases: 100% Controls: NR | Radiological (mpMRI) stage for cases:  T2a 8 (6%)  T2b 4 (3%)  T2c 71 (53.4)  T3a 38 (28.6%)  T3b 12 (9%)  Controls: NR | Gleason 6: 30 (22.6%)  Gleason 7: 77 (57.8%)  Gleason ≥8: 26 (19.5%) | NR |
| Roine | 2014 | Finland | Single centre urology outpatient department | Cases: median 63.5 (range 49-73)  Controls: median 67 (range 53-72) | NR | NR | Cases: mean 36.7 (range 2-18.2)  Controls: mean 3 (range 0.2-9) | NR | Pathological stage for cases:  pT2 27 (54%)  pT3 23 (46%) | Gleason 6: 9 (18%)  Gleason 7: 34 (68%)  Gleason ≥8: 7 (14%) | Cases: radical prostatectomy, controls: TURP |
| Taverna | 2022 | Italy | Multi-centre urology outpatient department | Cases: median 67  Controls: median 50.5 | NR | Controls: no family history  Cases: NR | Cases: range 2.5-44  Controls: range 0.1-2.2 | NR | Cases:  T1c: 72 (82%)  T2a 3 (3.4%)  T2b 1 (1.1%)  T2c 1 (1.1%)  T3a 3 (3.4%)  T3b 3 (3.4%)  T4 5 (5.7%)  Controls NR | NR | Cases: Prostate biopsy or incidental cancer on TURP |

Supplementary Figure 2: STARD Results

Supplementary Figure 3: Excluded Studies

Targeted at sarcosine(1-6)

Did not report sensitivity and specificity, or means to calculate them(7-12)

Did not use GC-MS or sensor(13-15)

Reported sensitivity and specificity in plasma but not urine(16)

Conference abstract(17)

1. Bianchi F, Dugheri S, Musci M, Bonacchi A, Salvadori E, Arcangeli G, et al. Fully automated solid-phase microextraction-fast gas chromatography-mass spectrometry method using a new ionic liquid column for high-throughput analysis of sarcosine and N-ethylglycine in human urine and urinary sediments. Anal Chim Acta. 2011;707(1-2):197-203.

2. Cavaliere B, MacChione B, Monteleone M, Naccarato A, Sindona G, Tagarelli A. Sarcosine as a marker in prostate cancer progression: A rapid and simple method for its quantification in human urine by solid-phase microextraction-gas chromatography-triple quadrupole mass spectrometry. Analytical and Bioanalytical Chemistry. 2011;400(9):2903-12.

3. Gao YH, Xu XJ, Song GX, Hu YM, Cheng HF. A Novel Derivatization Method for Separation of Sarcosine from Isobaric l-Alanine in Human Urine by GC-MS. Chromatographia. 2013;76(17-18):1181-6.

4. Jentzmik F, Stephan C, Miller K, Schrader M, Erbersdobler A, Kristiansen G, et al. Sarcosine in urine after digital rectal examination fails as a marker in prostate cancer detection and identification of aggressive tumours. Eur Urol. 2010;58(1):12-8; discussion 20-1.

5. Shamsipur M, Naseri MT, Babri M. Quantification of candidate prostate cancer metabolite biomarkers in urine using dispersive derivatization liquid-liquid microextraction followed by gas and liquid chromatography-mass spectrometry. J Pharm Biomed Anal. 2013;81-82:65-75.

6. Wu H, Liu TT, Ma CG, Xue RY, Deng CH, Zeng HZ, et al. GC/MS-based metabolomic approach to validate the role of urinary sarcosine and target biomarkers for human prostate cancer by microwave-assisted derivatization. Analytical and Bioanalytical Chemistry. 2011;401(2):635-46.

7. Bannaga AS, Kvasnik F, Persaud KC, Arasaradnam R. Differentiating cancer types using a urine test for volatile organic compounds. J Breath Res. 2021;15(1).

8. Barocas DA, Motley S, Cookson MS, Chang SS, Penson DF, Dai Q, et al. Oxidative stress measured by urine F2-isoprostane level is associated with prostate cancer. J Urol. 2011;185(6):2102-7.

9. Santonico M, Pennazza G, Asimakopoulos AD, Del Fabbro D, Miano R, Capuano R, et al. Chemical Sensors for Prostate Cancer Detection Oriented to Non-invasive Approach. Procedia Engineering. 2014;87:320-3.

10. Buszewska-Forajta M, Raczak-Gutknecht J, Struck-Lewicka W, Niziol M, Artymowicz M, Markuszewski M, et al. Untargeted Metabolomics Study of Three Matrices: Seminal Fluid, Urine, and Serum to Search the Potential Indicators of Prostate Cancer. Front. 2022;9:849966.

11. Yumba-Mpanga A, Struck-Lewicka W, Wawrzyniak R, Markuszewski M, Roslan M, Kaliszan R, et al. Metabolomic Heterogeneity of Urogenital Tract Cancers Analyzed by Complementary Chromatographic Techniques Coupled with Mass Spectrometry. Curr Med Chem. 2019;26(1):216-31.

12. Jiménez-Pacheco A, Salinero-Bachiller M, Iribar MC, López-Luque A, Miján-Ortiz JL, Peinado JM. Furan and p-xylene as candidate biomarkers for prostate cancer. Urol Oncol. 2018;36(5):243.e21-.e27.

13. Cook T, Ma YF, Gamagedara S. Evaluation of statistical techniques to normalize mass spectrometry-based urinary metabolomics data. Journal of Pharmaceutical and Biomedical Analysis. 2020;177.

14. Lee BY, Mahmud I, Marchica J, Derezinski P, Qi F, Wang FB, et al. Integrated RNA and metabolite profiling of urine liquid biopsies for prostate cancer biomarker discovery. Sci. 2020;10(1).

15. Guest C, Harris R, Sfanos KS, Shrestha E, Partin AW, Trock B, et al. Feasibility of integrating canine olfaction with chemical and microbial profiling of urine to detect lethal prostate cancer. PLoS One. 2021;16(2):e0245530.

16. Yu CW, Niu LF, Li L, Li T, Duan LM, He ZT, et al. Identification of the metabolic signatures of prostate cancer by mass spectrometry-based plasma and urine metabolomics analysis. Prostate. 2021;81(16):1320-8.

17. Bax C, Capelli L, Grizzi F, Prudenza S, Taverna G, Ieee, editors. A novel approach for the non-invasive diagnosis of prostate cancer based on urine odour analysis. IEEE International Symposium on Olfaction and Electronic Nose (ISOEN); 2022 May 29-Jun 01; Aveiro, PORTUGAL2022.
